# Evaluation and comparison of oral function after resection of cancer of the upper gingiva in patients who underwent reconstruction surgery versus those treated with a prosthesis

**DOI:** 10.1186/s12903-021-01709-7

**Published:** 2021-07-15

**Authors:** Yu Ohashi, Kiyoto Shiga, Katsunori Katagiri, Daisuke Saito, Shin-ichi Oikawa, Kodai Tsuchida, Aya Ikeda, Jun Miyaguchi, Takahiro Kusaka, Hiroyuki Yamada

**Affiliations:** 1 Division of Oral and Maxillofacial Surgery, Department of Oral and Maxillofacial Reconstructive Surgery, Iwate Medical University, Morioka, Japan; 2 Department of Head and Neck Surgery, Iwate Medical University, Yahaba, Japan; 3 Head and Neck Cancer Center, Iwate Medical University Hospital, Yahaba, Japan

**Keywords:** Oral cancer, Neoplasms, Gingival, Maxillofacial surgery, Free flaps, Maxillofacial prosthesis, Palatal obturator

## Abstract

**Background:**

We retrospectively analyzed the articulation, mastication, and swallowing function of patients who underwent reconstruction or used a prosthesis after resection of the upper gingiva.

**Methods:**

This study included patients who underwent resection of cancer of the upper gingiva from January 2014 to December 2018. Articulatory function was evaluated with Hirose’s conversational function evaluation criteria. Mastication function was evaluated with the Yamamoto’s occlusion table. Swallowing function was assessed with the MTF (Method of intake, Time, Food) score.

**Results:**

The mean articulatory function score was 8 points in the Reconstruction Surgery Group (RSG) and 8.8 points in the Prosthesis Group (PG). The mean mastication function score was 2.8 points in the RSG and 3.3 points in the PG. The mean swallowing function score was M3T4F4 in the RSG and M4T4F4.3 in the PG.

**Conclusions:**

The prosthesis depends on the remaining occlusal support area. Our study suggest that prosthesis is better indication when there is more than one occlusal support area.

## Background

When radical resection for advanced cancer of the upper gingiva creates an oronasal or oroantral fistula, patients experience dyslalia, dysmasesis, and dysphagia. The quality of life of these patients is impaired because of eating difficulties caused by dysmasesis and dysphagia. Dyslalia results in social difficulty; difficulty with speech and eating can result in avoidance of social situations [1]. To solve these problems, we generally perform a free flap and/or bone flap or apply a prosthesis after resection of carcinoma of the upper gingiva.

In many cases, a free fibula flap, free scapula flap, or free iliac flap is applied for reconstruction after resection of advanced gingival cancer [2]. Maxillary reconstruction with free flap provides abundant tissue for reconstruction, the freedom to orient, shape, and the ability for reconstruction to be performed as a single stage procedure [3]. Immediate reconstruction surgery has the advantage that the oral-to-maxillary and oral-to-nasal-cavity defects are closed in a single operation. Reconstruction with free bone flaps has advantages for masticatory function if an implant can be placed. However, disadvantage of flee flaps include longer surgical and recovery times with increased potential for complications compared with prosthetic obturation [4]. And many patients do not desire this treatment because of the increased burden of autologous tissue collection and the necessity of implant placement surgery to improve masticatory function.

Prosthesis has been common approach for treating maxillectomy defects. The advantage of this technique includes a shorter operation time, shorter postoperative hospital stay, and complete visualization of the maxillectomy cavity, which simplifies oncologic surveillance [5]. However, the prosthesis often takes several months to complete its shape and if there is no tooth to support the prosthesis, the prosthesis will not be stable and may not improve chewing function. And larger defects are harder to obturate as the prosthesis may be overly heavy and difficult or impossible to retain, particularly in partially or totally edentulous patients [6, 7].

Both methods have drawbacks. However, few reports have compared functional outcomes of free flaps and prostheses, and the choice of reconstruction method remains controversial [4, 8–11]. Therefore, in this pilot study we evaluated masticatory function, swallowing function, and articulation function after reconstruction performed with a free flap or a prosthesis for advanced maxillary cancer to determine criteria for method selection.

## Methods

Informed consent was obtained from all subjects. This study was approved by the Human Research Ethics Committee at the Iwate Medical University (ethics ID MH2020-210).

### Patients

Twelve consecutive patients who underwent resection of cancer of the upper gingiva followed by reconstruction with a free flap (skin flap and/or bone flap) or with prosthesis application from January 2014 to December 2018 were included in this study. Resection surgery resulted in oronasal or oroantral fistula in all patients. Although reconstructive surgery is the primary method of reconstruction after resection, it was decided to apply a prosthesis to the post-resection reconstruction method with the aim of regressing the operative time in the case of older age or complications of underlying disease.

### Methods

Maxillary defects were classified according to a maxillectomy classification [12] and the occlusion area after resection was classified according to Eichner’s Index [13].

Articulatory function was evaluated with Hirose’s conversational function evaluation criteria [14], which evaluate the patient’s ability to speak with family members and others. The attending physician conducted a questionnaire-guided interview with the patient and evaluated the patient on the basis of the results. This method uses a 5-point scale. Evaluation points from conversations with family (1–5 points) and from conversations with others who are not family members (1–5 points) are summed. Conversation function is evaluated according to the total score as excellent (8–10 points), moderate (5–7 points), or poor (≤ 4 points) (Table 1).

**Table 1:** Hirose's conversational function evaluation criteria

	(A)family	(B)other people
1. Can understand well	5 points	5 points
2. Sometimes does not know	4 points	4 points
3. Can understand if he/she knows a topics	3 points	3 points
4. Can sometimes understand	2 points	2 points
5. Can not understand at all	1 point	1 point
A + B		
Excellent: 10–8 points can talk everyday, talk on new topics		
MODERATE: 7–5 points conversation is possible if the topic is limited		
Poor: ≤ 4 social language life is difficult		

Hajime Hirose, Guideline of Head and Neck Cancer. Japan Society for Head and Neck Cancer, 3rd edition, 169, 2018

Mastication function was evaluated with the chewing efficiency judgment table (Yamamoto’s occlusion table) [15]. In this evaluation method, patients identify foods they can consume from a list, and the class of the relevant food is scored. Thirty-three types of food, including Japanese foods, are classified into six levels from 1 to 6, and the class of consumable foods is scored. Soup that can be consumed with an edentulous jaw is 1 point, whereas hard foods have a high score. If a high-scoring food can be consumed, chewing ability is high. The level at which mastication is possible is evaluated in a stepwise manner according to the chewing efficiency judgment table. The score of the level at which there is a food that can be consumed on the chewing efficiency judgment table is used for evaluation (Table 2).

**Table 2:** Yamamoto’s occlusion table

Score	Foods
1	Soup
2	Boiled rice, pudding, tofu
3	Rice, boiled fish, fish mince, tuna sashimi, skewers of eel
4	Steamed rice, roll boiled fish paste, kon-nyaku, sausage, ham, squid sashimi
5	Salami, beef steak, French bread, dry squid, millet brittle, scallop string, picked scallion, jellyfish vinegar, sea cucumber, vinegar dumpling, shellfish
6	Rice cake, peanut, rice cracker, pickled radish abalone

Yamamoto T. The posterior artificial teeth position used for complete dentures cross-bite case. Practice in Prosthodontics 1972;5:395–400, Modification

Swallowing function was assessed with the MTF classification [16]. This evaluation method allows easy evaluation of swallowing function in daily clinical settings. This method evaluates which foods and how much the patient can actually consume. The total score for the three items of Method of intake (M), Time (T), and Food (F) is calculated. Each evaluation item is scored from 1 to 5 points. Ingestible foods are categorized from A to E, then the number of food categories ingested is summed (Table 3).

**Table 3:** MTF score

1. Method of intake		2. Time of intake		3. Foods
Only tube feeding	1 point	Tube feeding	Nothing	A: water, tea
Combination tube feeding	2 points	MORE than 50 min	1 point	B: potage, rich liquid food
Invention of meal	3 points	40 min	2 points	C: jelly, paste food
Some restrictions	4 points	30 min	3 points	D: whole rice bowl, soft food
No limit	5 points	20 min	4 points	E: regular diet
		10 min	5 points	*Score the number of food groups available

Yasushi Fujiwara, et al. Journal of Otolaryngology of Japan 100: 1401–1407, 1997

Results of each evaluation method were used to compare the reconstruction surgery group (RSG) and the prosthesis group (PG). Statistical analyses were carried out using Mann–Whitney test.

## Results

This study included six men and six women; the mean age of patients was 65.8 years. T classifications included cT2 in three patients (25.0%), cT3 in one patient (8.3%), and cT4a in eight patients (66.7%). The operative procedure for the primary lesion was partial resection in eight patients (66.7%), subtotal resection in three patients (25.0%), and total resection in one patient (8.3%). The surgical procedure for reconstruction was fibula free flap in two patients (16.7%) and combined fibula free flap and anterolateral thigh free flap in two patients (16.7%). A prosthesis was applied in eight patients (66.7%).

Defect classification [12] in the RSG was 2b in one patient (8.3%) and 2c in three patients (25.0%). Defect classification in the PG was 2a in all eight patients (66.7%).

Eichner’s Index [13] scores in the RSG were B2 in one patient (8.3%), B3 in one patient (8.3%), C2 in one patient (8.3%), and C3 in one patient (8.3%). Eichner’s Index scores in the PG were B1 in one patient (8.3%), B2 in two patients (16.7%), B3 in two patients (16.7%), B4 in one patient (8.3%), and C2 in two patients (16.7%) (Table 4).

**Table 4:** Characteristics of the patients

Item scales	Total N (%)	N (%) of RSG	N (%) of PG
All patients	12 (100)	4 (33.3)	8 (66.7)
Gender			
Male	6 (50)	3 (25)	3 (25)
Female	6 (50)	1 (8.3)	5 (60)
Age			
Mean	65.8	58.5	69.5
Range	41–82	41–71	58–82
40–49	1 (8.3)	1 (8.3)	–
50–59	2 (16.7)	1 (8.3)	1 (8.3)
60–69	5 (60)	1 (8.3)	4 (33.3)
70–79	2 (16.7)	1 (8.3)	1 (8.3)
80–89	2 (16.7)	–	2 (16.7)
T classification			
cT2	3 (25)	–	3 (25.0)
cT3	1 (8.3)	–	1 (8.3)
cT4a	8 (66.7)	4 (3.3)	4 (3.3)
Operation of primary			
Limited resection of maxilla	8 (66.7)	2 (16.7)	6 (50)
Subtotal resection of maxilla	3 (25.0)	2 (16.7)	1 (8.3)
Radical resection of maxilla	1 (8.3)	–	1 (8.3)
Method of reconstruction			
Fibra flap	2 (16.7)	2 (16.7)	–
Fibra flap + Anterolateral thigh flap	2 (16.7)	2 (16.7)	–
Prosthesis	8 (66.7)	–	8 (66.7)
Defect class			
1	–	–	–
2a	8 (66.7)	–	8 (66.7)
2b	1 (8.3)	1 (8.3)	–
2c	3 (25.0)	3 (25.0)	–
3a	–	–	–
3b	–	–	–
3c	–	–	–
4a	–	–	–
4b	–	–	–
4c	–	–	–
Eichiner’s Index			
B1	1 (8.3)	–	1 (8.3)
B2	3 (25)	1 (8.3)	2 (16.7)
B3	3 (25)	1 (8.3)	2 (16.7)
B4	1 (8.3)	–	1 (8.3)
C1	–	–	–
C2	3 (25)	1 (8.3)	2 (16.7)
C3	1 (8.3)	1 (8.3)	–

**Fig. 1:**
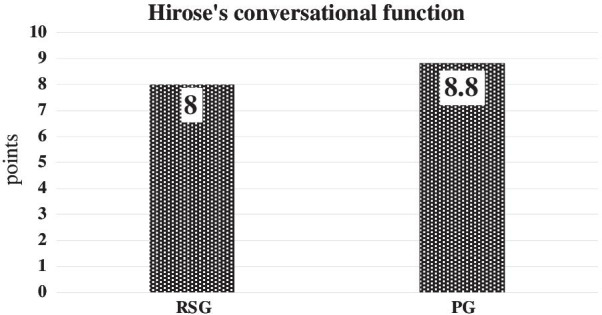
Hirose’s conversational function scores. The articulatory function score is slightly better un the PG patients than the RSG patients. There was no significant difference between these two groups (*p* = 0.29)

The mean articulatory function score was 8 points (min: 2, max: 10) in the RSG and 8.8 points in the PG; this difference was not significant (*p* = 0.29) (Fig. 1).

**Fig. 2:**
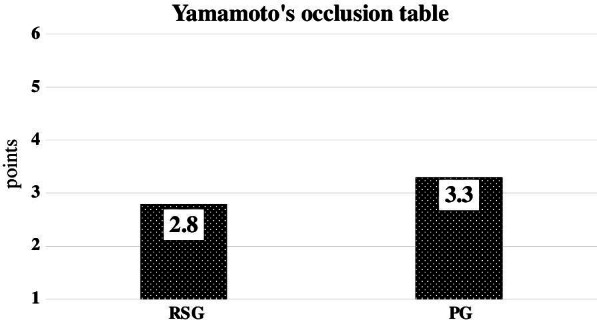
Yamamoto’s occlusion table scores. The mastication function score is slightly better in the PG patients than the RSG patients. There was no significant difference between these two groups (*p* = 0.60)

The mean mastication function score was 2.8 points (min: 1, max: 6) in the RSG and 3.3 points in the PG. This difference was not significant (*p* = 0.60) (Fig. 2).

**Fig. 3:**
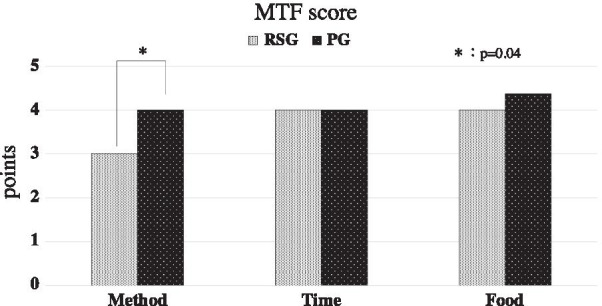
MTF scores. The swallowing function score is significantly better in the PG patients than the RSG patients in the category of food-intake method (*p* = 0.04). However, there was no significant difference between groups in intake time (*p* = 1.00)

The mean swallowing function score was M3T4F4 (min: M1T0F1, max: M5T5F5) in the RSG and M4T4F4.3 in the PG. In the category of food-intake method, patients in the PG ingested in a significantly more normal fashion than those in the RSG (*p* = 0.04). However, there was no significant difference between groups in intake time (*p* = 1.00) or in food groups (p = 0.58) (Fig. 3).

## Discussion

After resection of advanced carcinoma of the upper gingiva, there were two common methods: applying an obturator or performing a reconstructive surgery using a free flap (and/or bone flap). Reconstruction with an obturator has been reported to provide good quality of life [17, 18]. However, obturators can allow food leakage during swallowing [1] and there may be a decrease in feeling of attachment as a result of dry mouth after radiation treatment if the patients underwent postoperate radiotherapy [9]. It is said that prosthesis is not appropriate due to heavy weight [4, 6]. In contrast, reconstructive surgery using osteocutaneous free flaps (bone flaps) is reported to preserve significant function in the mid-face [19]. This procedure is thought to be highly effective [20], but has the drawback of increasing operation time [4]. In addition, occlusal reconstruction with placement of a dental implant after free bone flap reconstruction lengthens the treatment period, increases the surgical invasiveness, and requires more visits to clinics for treatment. In patients with advanced cancer of the upper gingiva, occlusal reconstruction with a dental implant is ideal after reconstruction of the maxilla with a free bone flap. However, postoperative radiation therapy or radiation chemotherapy is sometimes necessary. In these cases, the patient often does not want the additional visits required after reconstruction surgery. Although each treatment has unique advantages and disadvantages and there have been various reports on each treatment, Maurio et al. [4] reports that extensive maxillectomy defects have better functional outcome with free flap. On the other hand, there are reports that there is no difference in oral function between PG and RSG [8, 10, 11]. Oral functions including mastication, swallowing, and articulation, and maximum restoration of these functions is important. We believe that while aesthetic recovery is important, recovery of oral functions is directly linked to quality of life. Therefore, to clarify whether it is better to apply an obturator or to perform a reconstructive surgery with a free flap, we retrospectively examined the records of patients treated at our facility and evaluated their masticatory function swallowing function, articulatory function and so on. The purpose of this study was to determine treatment selection criteria by evaluating swallowing function and articulation function. Our study indicated that the size of the primary tumor and the occlusal support area influenced the functional evaluation. In this study, the RSG had four cT4a cases, whereas the PG had three cT2 cases, one cT3 case, and four cT4a cases. According to this classification, the PG tended to retain an occlusal support area, which we consider slightly more favorable.

There was little difference between groups in articulatory function, but function tended to be slightly better in the PG than in the RSG. However, some patients with a prosthesis were unable to converse when the prosthesis was removed at night, and a marked decline in function in daily life was a problem. In contrast, patients in the RSG were able to speak at any time, which is an advantage of surgical reconstruction.

Mastication function did not sufficiently recover in either group. It is probable that in the RSG only soft tissue reconstruction was performed in some cases and implant restoration was not performed even in some cases with hard tissue reconstruction. Patients in the PG tended to have good mastication function. If an implant prosthesis can be placed, mastication function is expected to improve. However, this approach increases the number of surgeries and the invasiveness of the procedures. In addition, some patients require prompt surgery to treat the underlying disease and do not have time to carefully consider treatment options. Indications need to be determined based on conditions such as the patient’s age, general condition, and the curability of the underlying disease.

With regard to swallowing function, a properly manufactured prosthesis can create a more natural environment in the oral cavity during the oral stage of eating and swallowing. An environment that promotes easy swallowing can be created during the prosthesis preparation process and with fine adjustments after completion. In contrast, with reconstruction surgery, because the formation of the intraoral environment is limited to the intraoperative period, it is difficult to create ideal conditions for restoring swallowing function.

We found no significant difference between the groups in most functional evaluations, although the PG tended to have relatively better functional outcomes. The PG had significantly better results in the method of intake; we think this difference is related to better swallowing function resulting from better marginal fitting of the prosthesis. The RSG did not have ideal occlusal recovery. These results indicate that occlusal reconstruction with a dental implant is necessary for better outcomes after reconstruction surgery. In both groups, masticatory function was markedly reduced. Because use of a prosthesis depends on the remaining occlusal support area, if this area is small, masticatory function may not recover. If a dental implant can be placed, masticatory function is expected to recover. When deciding whether to perform reconstruction surgery or to apply a prosthesis, it is important to focus on the occlusal support area after resection as well as the maxillary defect area, to consider the advantages and disadvantages of each method, and to fully consult with the patient and related medical staff. In patients with advanced maxillary cancer who do not have much time to prepare for surgery and in environments where reconstructive surgery is not possible, a prosthesis may be considered as a first choice. Our study suggest that prosthesis tend to have better oral function for Eichner B3. In addition, if radical resection is possible, patients understand the procedure, and there is no problem with patient age, underlying disease, or waiting time for surgery, reconstructive surgery and functional restoration with implant prosthesis is preferable.

A limitation of this study was due to the study design. This pilot study was a retrospective review of small number of the patients who underwent maxillectomy in our institutions. Therefore, an organized prospective study is needed to evaluate the treatment results of the patients who undergo maxillectomy. If possible, multi-institutional prospective study is necessary to clarify the criteria of the selection of functional treatment for the patients who undergo maxillectomy.

## Conclusions

There was no significant difference between the two treatment groups in most functional evaluations, although the PG tended to have relatively better outcomes than the RSG. The PG had significantly better results in the method of food intake. This finding may be related to better swallowing function resulting from better marginal fitting of the prosthesis. Occlusal reconstruction with a dental implant is necessary to improve outcomes after reconstruction surgery. Our study suggest that prosthesis is indicated for Eichner B3. If radical resection is possible, the patient understands the procedure, and there are no problems with patient age, underlying disease, or waiting time for surgery, reconstructive surgery and functional restoration with implant prosthesis is preferable.

## Data Availability

All data used and/or analyzed during this research are available from the corresponding author on reasonable request.

## Abbreviations

RSG: Reconstruction Surgery Group
PG: Prosthesis Group
